# A Novel Request

**Published:** 1914-06-20

**Authors:** 


### A Novel Request.

In the " agony column " of the *Times* of June 17 appears an advertisement which runs as follows:

> Wanted, 50 Malaria Patients to Undergo Free Curative Treatment under supervision of West-End physicians. No quinine or arsenic administered; relapsing cases or patients returning to malaria-stricken districts preferred. Applicants address to A. B., care of a firm of solicitors.

We have no information whatever as to the " curative treatment " thus advertised, nor of the " West-End physicians " who are to " supervise " it, nor of the individual who conceals his identity under the initials A. B. The latter may or may not be a member of the medical profession. If he is, we object very decidedly to his reticence and his anonymous request for clinical material; if he is not, the West-End physicians are in the position of " covering " an unqualified practitioner. In either case we say without hesitation that this hole-and-corner method of testing new treatments is not one which can obtain the approval of the medical profession, or is at all likely to promote the public weal or any other useful purpose.

If there is any reasonable ground for supposing that this " curative treatment," whatever it may be, is of use in combating malaria, the evidence in favour of that view should be published. Such a course has many advantages. In the first place, it enables practitioners of all races to test the method, if they wish to do so, and so leads to a speedier and fairer verdict upon the value of the remedy. Secondly, the name and reputation of the person or persons who have conducted the preliminary tests or researches enable the medical profession to form some idea of whether the proposals put forward are to be taken seriously or not. Thirdly, the nature of the experimental evidence, or of the ratiocination upon which the hope of benefit is based, can be examined and assessed.

We see no reason why this particular disease, malaria, should be exempted from the salutary custom, and many reasons why it should not. The principal of these is that the diagnosis of malaria is an intricate and difficult business. Everyone who has been in the tropics and falls ill with pyrexia as a symptom promptly labels himself malaria and overdoses himself with quinine. If he gets well, he concludes that he is quite capable of treating himself without medical advice; if he does not, instead of drawing the (probably correct) inference that his disease is something other than malaria, he experiments on himself with all sorts of patent remedies, which may or may not contain quinine. Such a man, in the present case, is only too likely to offer himself as clinical material for A. B., with dire results.

There remains the position of the " West-End physicians." Those who remember the action which a certain consultant brought (successfully) against a nobleman two or three years ago will deprecate very earnestly any repetition of the arrangements then disclosed by which a medical man associated himself with an unqualified practitioner in the conduct of research. The profession of medicine cannot too strongly, or too immediately, express its disapproval of A. B.'s methods, unless a much nearer approach is made to that wholesome publicity in the professional press which is the proper and well-recognised system of promoting research.

